# Technical results from a trial of the FREO_2_ Low-Pressure Oxygen Storage system, Mbarara Regional Referral Hospital, Uganda

**DOI:** 10.1371/journal.pone.0248101

**Published:** 2021-03-09

**Authors:** David Peake, James Black, Elias Kumbakumba, Sheillah Bagayana, Celestine Barigye, Peter Moschovis, Ivan Muhumuza, Frank Kiwanuka, Patrick Semata, Kevin Rassool, Bryn Sobott, Roger Rassool

**Affiliations:** 1 FREO_2_ Foundation Australia Ltd, Melbourne, Australia; 2 School of Physics, Faculty of Science, The University of Melbourne, Parkville, Australia; 3 Mbarara Regional Referral Hospital, Mbarara, Uganda; 4 Mbarara University of Science and Technology, Mbarara, Uganda; 5 Massachusetts General Hospital, Harvard Medical School, Boston, MA, United States of America; University of Notre Dame Australia, AUSTRALIA

## Abstract

Increased access to reliable medical oxygen would reduce the global burden of pneumonia. Oxygen concentrators have been shown to be an effective solution, however they have significant drawbacks when used in low-resource environments where pneumonia burden is the heaviest. Low quality grid power can damage oxygen concentrators and blackouts can prevent at-risk patients from receiving continual oxygen therapy. Gaps in prescribed oxygen flow can result in acquired brain injuries, extended hypoxemia and death. The FREO_2_ Low-Pressure Oxygen Storage (LPOS) system consists of a suite of improvements to a standard oxygen concentrator which address these limitations. This study reports the technical results of a field trial of the system in Mbarara, Uganda. During this trial, oxygen supplied from the LPOS system was distributed to four beds in the paediatric ward of Mbarara Regional Referral Hospital. Over a three-month period, medical-grade oxygen was made available to patients 100% of the time. This period was sufficient to quantify the ability of the LPOS system to deal with blackouts, maintenance, and an unscheduled repair to the LPOS store.

## Introduction

Oxygen is essential in the treatment of hypoxaemic illness in paediatric patients, reducing mortality and sequelae [1, 2]. However in low resource settings, reliable oxygen supplies are rarely available in health facilities [3]. Concentrators and cylinders are mature technologies, however there are critical issues limiting their use–primarily with the longevity and reliability of concentrators in settings with unreliable electricity [4], and with the cost of acquiring, filling and transporting cylinders [5]. To increase access to reliable oxygen supplies in low resource settings, a Low-Pressure Oxygen Storage (LPOS) system [6] has been designed to mitigate the major limitations of concentrators and cylinders in low resource settings.

The LPOS system comprises a power conditioner, oxygen storage utilising low-pressure reservoirs, pneumatic switch, and low-cost oxygen distribution to individual patients. In combination with a standard concentrator and a backup cylinder, these devices are intended to improve reliability of oxygen supply whilst reducing the cost of oxygen for the health facility. By buffering short power outages using the local low-pressure storage and switching to more expensive high-pressure storage (cylinders) during extended outages, the LPOS system augments existing concentrator technology to provide reliable oxygen access to patients. This article presents key findings from the first trial of the LPOS system in clinical use in the paediatric ward of a health facility in Africa.

## Methods

### Study setting

Mbarara Regional Referral Hospital is the major public referral hospital for Western Region, Uganda. The paediatric ward has 70 inpatient beds [7] and routinely uses a combination of mains-powered oxygen concentrators and oxygen cylinders to provide oxygen to hypoxaemic inpatients. A backup electricity generator is available to the paediatric ward but power interruptions are still frequent. The limited number of available cylinders are expensive and need to be manually exchanged by a technician. The hospital has a small engineering and maintenance group, with skills in biomedical engineering, electrical systems and plumbing.

### Patient and public involvement

Due to the technical nature of the work, patients or the public were not involved in the design, conduct, reporting, or dissemination plans of this research. As the system moved towards large scale production, we are including the public (ward staff and maintenance personnel) in the continuing user-centric design process.

### Low Pressure Oxygen Storage (LPOS) system components

**Fig 1:**
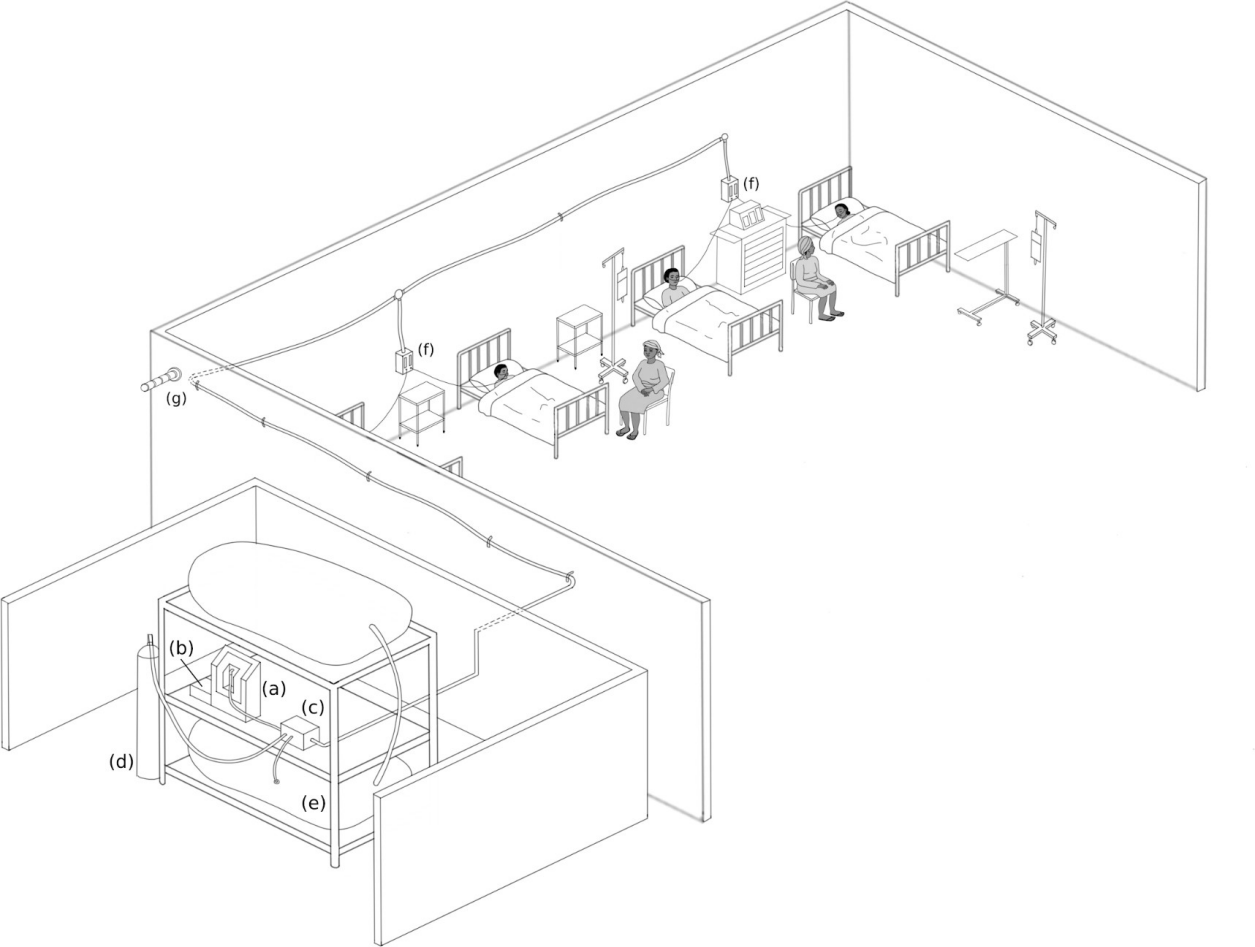
Relative location and components of the LPOS system. The oxygen concentrator (a), PROTECT (b), Prioritizer (c), backup cylinder (d) and low-pressure local storage (e) are situated outside of the ward. The oxygen is piped via a low-cost oxygen distribution system to a volumetric flow meter (f) at each bed. A stack lamp (g) representing the status of the system is situated in the nurse’s office. Image created by Isabella Anderson, inspired by a similar illustration created by David Woodroffe [8].

As shown in Fig 1, the LPOS system combines a suite of devices designed to address context specific challenges.

#### PROTECT

The electrical requirements of oxygen concentrators recommended for low-resource settings, such as the AirSep *Elite* and DeVilbiss *525KS* [9], is constant 230 VAC at 50 Hz [10]. However, health clinics in low resource settings cannot guarantee access to reliable electricity at this voltage [11]. Consequently, the warranties are voided and longevity reduced. In order to ensure the long-term viability of the oxygen system, FREO_2_ developed a power conditioning system, PROTECT.

During the research and development phase of PROTECT, a wide range of commercially available off-the-shelf voltage stabilisers were assessed for compatibility with AirSep Elite concentrators. Findings revealed that the large current required by the air compressors during start-up caused the off-the-shelf voltage stabilisers to fail. PROTECT has been designed to address this gap by conditioning the power to meet the specific requirements of oxygen concentrators. Functions include voltage stabilisation for inputs between 180 VAC to 260 VAC, automatic shut-down outside that range, and controlled restarts to avoid strain on the compressor created by rapid power cycling.

#### Oxygen concentrator

The concentrator used was an off-the-shelf AirSep® NewLife® Intensity designed to produce up to 10 litres per minute of 87% - 95.5% oxygen on 230 VAC electricity.

#### LPOS store

**Fig 2:**
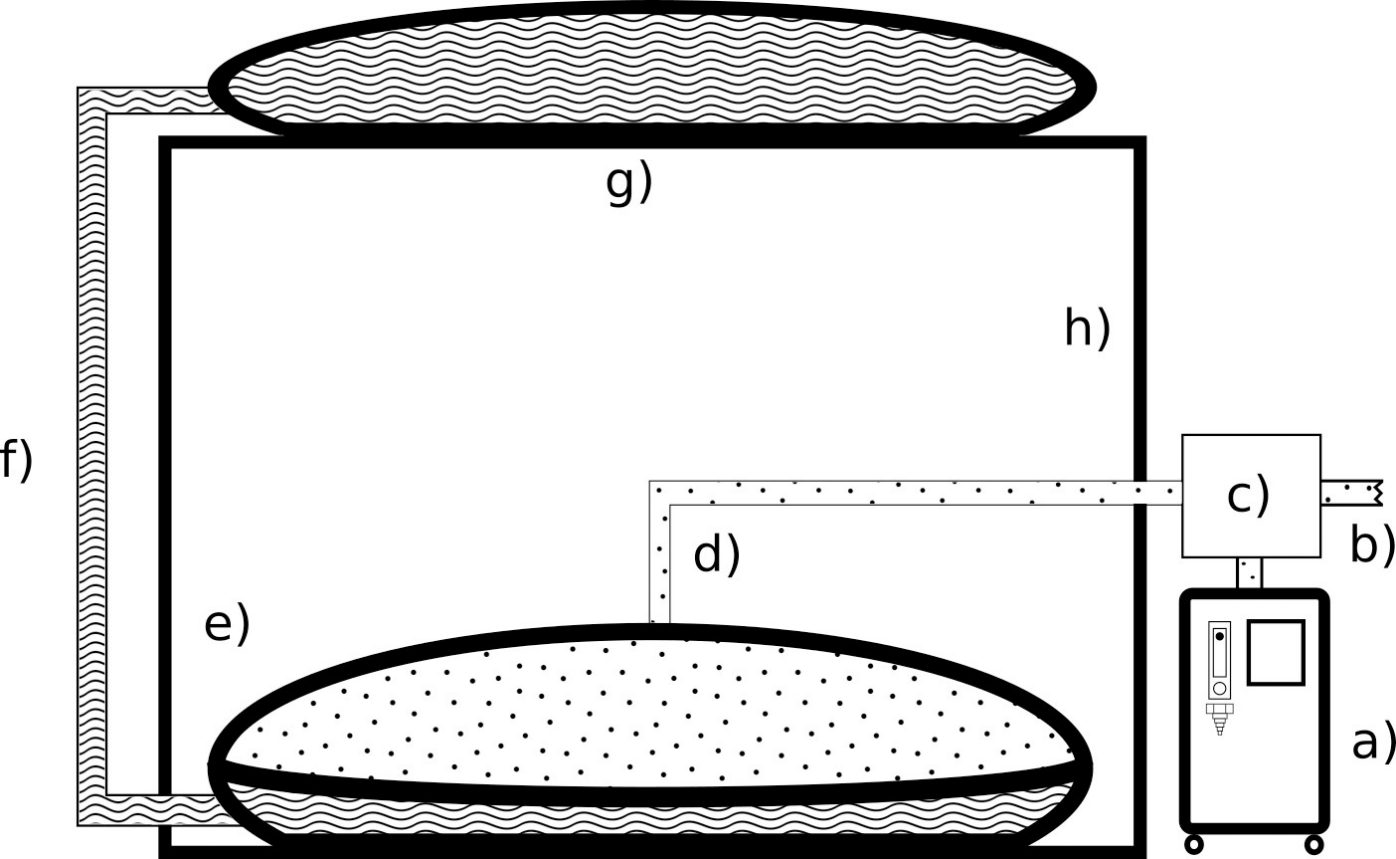
The FREO_2_ Low-Pressure Oxygen Storage system. a) Oxygen Concentrator, b) Flow to patient, c) Prioritizer, d) Oxygen flow to and from LPOS, e) The lower PCV bag which has two airtight bladders internally, f) water connection from the lower water bladder to the upper water bladder, g) the upper PVC bag including one water bladder, h) mechanical frame. Not shown: emergency oxygen cylinder.

The LPOS Store has been previously described in detail [6]. Fig 2 shows a diagrammatic representation of the LPOS store. Briefly, LPOS comprises two internal chambers, which allow for the concurrent storage of a fluid and a gas. The fluid section of the lower reservoir is initially filled to capacity with water. A hose links this section to the empty upper reservoir, which is positioned at a predefined height.

During operation, the oxygen concentrator is set to maximum output and oxygen that exceeds patient demand automatically flows to LPOS. As oxygen enters the gas section of the lower reservoir, it forces the water in the fluid section into the upper reservoir. As the water and oxygen are contained in separate airtight bags, the water does not contact the oxygen or evaporate. In the event of a power failure, the weight of the water previously displaced into the upper reservoir provides the force to drive the stored oxygen to the patient, without the need of a pump or battery. This approach is concentrator agnostic and does not require any modification of the concentrator.

The LPOS store installed for this trial holds approximately 240 L of oxygen which can provide for four hours of oxygen for a child prescribed 1 LPM of oxygen. The store scales well, with a 1000L prototype currently being investigated.

#### Prioritizer

Prioritizer is a pneumatic switch that automatically routes the lowest cost oxygen to patients. If the power is on, oxygen flows from the concentrator. If the power is off, oxygen flows from LPOS. If the power is off and LPOS is empty, then flow from the back-up cylinder is automatically activated. No user intervention is required to change between oxygen sources.

#### Backup cylinder

Standard oxygen cylinders were used to maintain oxygen flow to patients during extended power outages. The hospital engineering staff regularly checked the cylinder pressure to minimise the risk of running out of cylinder oxygen.

#### Stack lamp

The status of the system is displayed to nursing staff via a traffic light-style lamp. It has three states: Green indicates that the concentrator is on, Orange indicates that the LPOS is in use and Red signifies that the backup cylinder has been enabled.

#### Low pressure distribution

Distributing the oxygen to patients is relatively straightforward but can be prohibitively expensive. For example, a study in the Gambia indicated a cost of USD$11, 000 for a 20-bed 6-cylinder bank system [12]. Further, leaking can cause between 10 and 80% of stored oxygen to be wasted [12]. By operating at low pressures, copper piping can be replaced with polyurethane tubing. Medical grade tubing is currently sourced from APS Medical, based in Malaysia, at a cost of USD$0.41 per meter with an expected cost for four beds per ward of approximately USD$200 including the wall mounted outlets.

#### Location

As described in Fig 1, the LPOS system was installed in a prepared alcove, outside of the paediatric ward. By installing the system external to the ward and providing oxygen to the patients via the oxygen distribution system, the FREO_2_ LPOS system can provide reliable oxygen without occupying valuable ward space. This approach also enables health care workers to focus on the needs of the patient instead of specific concentrators or cylinders.

### Technical phase

In preparation for the clinical trial, a two month on-site technical phase was undertaken. During this technical phase, the pressure in the oxygen reservoir was monitored using an NPX USA MPX4250 pressure sensor, and the oxygen flow rates from the concentrator, into and out of the LPOS store, and to the simulated patient were monitored using Sensirion SFM3000 flow meters. Oxygen concentration was monitored with a Longfian Scitech JAY-120 oxygen analyzer. Each of these sensors was calibrated against laboratory standards and linked via an I^2^C serial bus. The sensors were polled at 1.25 Hz by a specialized data acquisition system. The data were routinely logged to solid state drives and uploaded to a remote server to enable real-time remote monitoring. A backup oxygen cylinder was connected to the Prioritizer subsystem, and formal tests made of the performance of the system under simulated power failure conditions at different oxygen flow rates.

### Clinical phase

Prior to supplying oxygen to patients, the technical phase data was analysed to confirm that the system was performing according to specification. Volumetric Flow Meters (VFMs) were installed to four designated beds and oxygen made available to the ward staff for use. No changes were made in the process of diagnosis of pneumonia or hypoxaemia, the criteria for the use of oxygen, nor any other aspect of treatment. The flow to each child could be individually adjusted between zero and two litres per minute to meet the needs of the patient.

### Ethical considerations

Ethics approval was granted (Approval Number 11/04-17) by the Human Research Ethics Committee of Mbarara University of Science and Technology and the Uganda National Council of Science and Technology (HS 2361). The nature of the trial was explained to the parents of all prospective patients and written informed consent was obtained prior to treatment. Although no caregivers refused LPOS oxygen, if consent was not given the child was admitted to a different bed and received standard care using a combination of piped cylinder oxygen and standard oxygen concentrators. All components of the system were the same as already routinely in use in the ward, or formally approved as medical or food grade by the United States Food and Drug Administration (FDA). The trial was funded by FREO_2_ Foundation Australia.

## Results

### Technical assessment

The clinical field trial was undertaken for a period of 3 months starting from 17/07/2018 through to 17/10/2018 inclusive. Data acquired during this trial period is publicly available at [13]. During this time, the paediatric ward experienced a total of 215 power outages, accounting for 5.7% of the total study time. Of these, 106 were less than 1.5 minutes duration and the PROTECT unit intervened and interrupted the power line to the concentrator.

**Fig 3:**
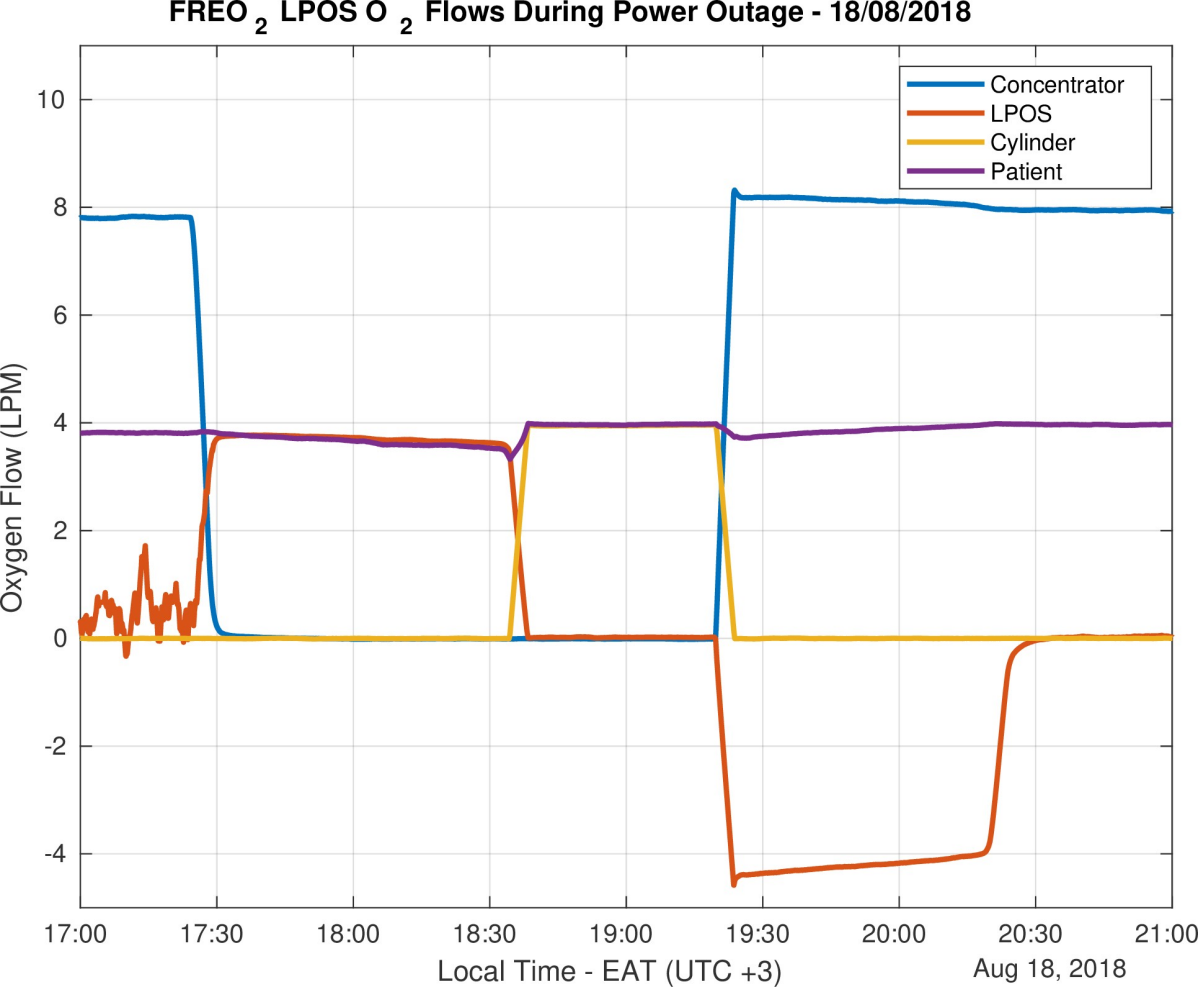
The flow rates (in litres per minute) in each component of the LPOS system during a prolonged power cut demonstrating uninterrupted flow to the patient. On 18/09 the power was interrupted for two hours, and the oxygen was supplied by LPOS to maintain patient flow. As the store was depleted, Prioritizer automatically recruited oxygen from the cylinder.

Of the remaining 109 events, the LPOS Store provided all required oxygen for 90 outages greater than 1.5 minutes. As shown in Fig 3, during the remaining 29 outages the Prioritizer automatically switched to the backup cylinder. Four of the outages were due to scheduled concentrator maintenance during which the LPOS store provided oxygen. On one occasion the backup cylinder provided oxygen while a repair was made to the LPOS store.

Table 1 provides the summary statistics for flows towards all included patients from the concentrator, LPOS Store and cylinder for the duration of the trial. During the trial, oxygen was required for admitted patients 97.8% of the time, and oxygen was available from the LPOS system 100% of the time, whether or not there was patient demand, with no interruptions or shortfalls.

**Table 1:** Summary statistics for the concentration, pressure and flow of oxygen supplied by the LPOS system during the clinical phase of the trial.

	Concentrator flow (SLPM)	LPOS^§^ flow to patients (SLPM)	Cylinder flow (SLPM)	Patient flow (SLPM)
**10**^**th**^ **percentile**	7.7	1.6	2.3	1.6
**Mean**	8.0	3.5	3.6	3.5
**Median**	8.1	3.4	3.0	3.4
**90**^**th**^ **percentile**	8.4	5.5	5.6	5.5
**Sum (L)**^**‡**^	436,176	15,070	13,145	464,391
**Flow proportion (%)**	93.9	3.2	2.8	100
**Time proportion (%)**^**†**^	94.3	2.0	2.8	97.8

All measurements are in standard litres per minute (SLPM). ‘Concentrator flow’ means the total output of the concentrator. The other flows are those delivered to the patients from each component.

^**§**^Recharge flow into the LPOS Store is not included.

^**‡**^Total flow delivered to patients from each source, in litres.

^**†**^The proportion of the total time that there was flow to the patients from this source, as percentage.

In the table the sum of the proportions of time during which flow came from each component is not equal to the total proportion of time that patients received oxygen. This is due to brief periods in the change-over from one to another during which two sources were supplying oxygen simultaneously.

### Clinical assessment

During the clinical trial, 56 children were treated with oxygen from the LPOS system. This represents an average of 8,293 litres per child, and an average of approximately 39.5 hours of oxygen treatment per child.

Four deaths were recorded among the children receiving LPOS oxygen: one was assessed by the attending doctors as due to complications of severe pneumonia, one as tuberculosis plus malnutrition, one as septicaemia and gastroenteritis, and one as pyloric stenosis with hyponatraemia and severe hypokalaemia. Detailed analysis of the data confirmed that there were no power interruptions of more than two hours, and no break in oxygen availability during any of these children’s admissions. They all received primarily concentrator oxygen via the LPOS system, with supplemental oxygen via the LPOS Store. Only one received backup cylinder oxygen, in three separate periods, with a total of two and a half hours over a 20 day admission.

## Discussion

The LPOS system provided an uninterrupted supply of up to 8 litres per minute (LPM) of oxygen to four paediatric beds during the three-month trial of continuous monitoring. During that time there were many interruptions to concentrator function, mostly due to unscheduled cuts in grid electricity. Although the majority were less than 15 minutes, some lasted more than six hours.

The hospital engineering department provided routine maintenance for the concentrator and rectified minor mechanical failures occurring during the trial. As anticipated, most of the oxygen was provided by the standard AC-powered oxygen concentrator, with about 3.3% automatically provided from the LPOS Store and 2.8% from the backup cylinder. No intervention was required by the ward clinical staff at any point. No other system offering 100% reliable oxygen without clinical staff intervention is available to the hospital.

The cost of this prototype LPOS system was USD$2,300—including the cost of concentrator, LPOS store, low-pressure piping to the ward, volumetric flow meters and installation. Based on the local price of electricity of USD 30 c/kW.h, and assuming an average concentrator power consumption of 590 W the cost of electricity required during the trial was USD$369. During the trial, two cylinders of oxygen at a cost of USD$20, excluding transport, were required to cover periods when the LPOS store was empty. The cost of regularly cleaning filters and simple maintenance of the system is estimated to be USD$120 per year. Therefore, the overall running cost is estimated to be USD$439 per quarter to supply up to 8 LPM of reliable oxygen spread across four paediatric beds. If the costs measured during the trial are approximately constant during the year this would lead to an annual cost of USD$1756.

As tabled above, the LPOS system delivered 464,391 litres of oxygen to the patients, offsetting the purchase of 75 cylinders at a cost of USD$1500, excluding transport, per quarter. Therefore, over the trial period the LPOS system delivered a net savings of USD$1061, which demonstrates a potential payback period of less than 1 year. Cost-savings will be significantly larger for remote clinics, where the transport cost of cylinders can exceed the cost of the oxygen itself [14].

Storing the oxygen at low pressure requires a larger amount space than storing it at high pressure in a cylinder. The installation site at Mbarara was a disused alcove external to the paediatric ward which we modified with a locking gate. As the system has been designed to be not require attention from nursing staff, it does not need to be located within the ward and can be installed in unused storage areas, external alcoves or even in purpose-built sheds. Find space is an issue for low-pressure oxygen storage but we found that available space is common in the environment in which LPOS is designed to operate.

LPOS is designed to operate in a “grey-power” environment where mains power is available but not reliable. In areas where mains power is not available or is of such poor quality that it prevents the concentrator from filling the oxygen store, solar-powered concentrators are another solution. There are currently trials underway in Uganda investigating the effectiveness of solar powered concentrators [15] and FREO_2_ is also in the prototype stage of testing a novel solar powered oxygen concentrator. A combination of mains powered, and solar powered systems will be necessary to increase access to medical oxygen to all low-resource environments. An LPOS store attached to a solar-powered oxygen concentrator can also increase the efficiency of the solar concentrator by turning off the concentrator once the oxygen storage is full, effectively duty cycling the concentrator. This combination of technologies could reduce the overall cost of a solar powered oxygen concentrator system.

In order for an innovation such as LPOS to be effectively taken up in low-resource environments, a clear economic advantage must be demonstrated. A full economic analysis was outside the scope of this trial but remains a high priority for future work. We have continued to measure important economic parameters such as electricity and maintenance costs in preparation for a deeper analysis.

The LPOS system has now continuously operated for one year and demonstrates robustness and reliability in day-to-day clinical use in the paediatric ward of a busy African hospital. It is now in routine use in the same ward and is ready for trials in a wider range of health facilities, including district hospitals and sub-district health centres.

## Conclusion

This study has shown that the addition of the LPOS system allows for long term uninterrupted oxygen delivery from a concentrator, even through extended power outages. The system supplied up to 8 LPM of oxygen to four beds in the Mbarara paediatric ward over the clinical trial period with 100% oxygen availability. Relative to oxygen provision solely from cylinders, a net saving of USD$1061 and a payback period of less than 1 year was demonstrated. By adding innovations which condition poor power supply and store excess oxygen at low pressures, the LPOS system increases the effectiveness of a common off-the-shelf oxygen concentrator, increasing access to life-saving oxygen therapy.
